# Evidence for Resistance to Coagulotoxic Effects of Australian Elapid Snake Venoms by Sympatric Prey (Blue Tongue Skinks) but Not by Predators (Monitor Lizards)

**DOI:** 10.3390/toxins13090590

**Published:** 2021-08-24

**Authors:** Nicholas J. Youngman, Joshua Llinas, Bryan G. Fry

**Affiliations:** 1 Venom Evolution Lab, School of Biological Sciences, University of Queensland, St Lucia, QLD 4072, Australia; n.youngman@uq.edu.au; 2 Unusual Pet Vets, Jindalee, QLD 4074, Australia; josh@unusualpetvets.com.au

**Keywords:** coagulotoxins, plasma, *Pseudechis*, *Pseudonaja*, resistance, *Tiliqua*, *Varanus*

## Abstract

Some Australian elapids possess potently procoagulant coagulotoxic venoms which activate the zymogen prothrombin into the functional enzyme thrombin. Although the activity of Australian elapid prothrombin-activators has been heavily investigated with respect to the mammalian, and in particular, human clotting cascades, very few studies have investigated the activity of their venom upon reptile plasmas. This is despite lizards representing both the primary diet of most Australian elapids and also representing natural predators. This study investigated the procoagulant actions of a diverse range of Australian elapid species upon plasma from known prey species within the genera *Tiliqua* (blue tongue skinks) as well as known predator species within the genera *Varanus* (monitor lizards). In addition to identifying significant variation in the natural responses of the coagulation cascade between species from the genera *Tiliqua* and *Varanus* relative to each other, as well as other vertebrate lineages, notable differences in venom activity were also observed. Within the genus *Tiliqua*, both *T. rugosa* and *T. scincoides* plasma displayed significant resistance to the procoagulant activity of *Pseudechis porphyriacus* venom, despite being susceptible to all other procoagulant elapid venoms. These results indicate that *T. rugosa* and *T. scincoides* have evolved resistance within their plasma to the coagulotoxic venom activity of the sympatric species *P. porphyriacus*. Other venoms were able to activate *Tiliqua* prothrombin, which suggests that the lessened activity of *P. porphyriacus* venom is not due to modifications of the prothrombin and may instead be due to a serum factor that specifically binds to *P. porphyriacus* toxins, as has been previously seen for squirrels resistant to rattlesnake venom. In contrast, none of the predatory lizards studied (*Varanus giganteus*, *V. mertensi* and *V. varius*) demonstrated resistance to the venom. This suggests that the mechanical protection afforded by thick osteodermic scales, and prey handling behaviour, removes a selection pressure for the evolution of resistance in these large predatory lizards. These results therefore reveal differential interactions between venoms of snakes with sympatric lizards that are on opposite sides of the predator–prey arms race.

## 1. Introduction

Venom is a key evolutionary innovation underpinning the explosive radiation of advanced snakes. Previous research has indicated that venom specificity and potency is closely associated with a species’ dietary composition [1,2]. Venoms of specialist predators are often evolutionary adapted for targeting their specific prey, whereas the toxins present in the venoms of generalist predators possess broader specificity to enable the subjugation of prey with varying physiologies [2]. This is a crucial aspect to consider when investigating venom activity, because the venoms’ effect upon the physiology of a single species may differ dramatically from its effect upon another species. Early studies on *Boiga irregularis* concluded it was non-venomous due to the lack of effects when tested on rodents [3]. However, subsequent tests showed that this species and other colubrid snakes were dramatically more potent upon the lizards or birds upon which they prey, than non-prey types such as rodents [4,5,6]. Similarly, a recent study upon the *Denisonia* genus identified that the known amphibian specialist *D. devisi* showed significant anticoagulant activity only upon amphibian plasma [7].

Venoms are evolutionarily selected to be prey-selective; there is a corresponding selection pressure for the evolution of resistance in prey and predators of venomous snakes. Such resistance may be accomplished in a myriad of ways. For example, two mechanisms of resistance to α-neurotoxins (steric hinderance and electrostatic repulsion) have been discovered, with both mechanisms involving modifications of the othosteric site of the nicotinic acetylcholine receptor α-subunit [8,9,10,11]. Resistance to blood-acting toxins in opossums, marsupials from the Americas which predate upon small species of snake but are predated upon by larger species, has occurred through modification of the targeted blood protein von Willebrand factor [12]. Modification in the blood-clotting factor fibrinogen from *Bothrops jararaca* has also been revealed to convey resistance towards thrombin-like enzymes [13,14]. Conversely, resistance in squirrels to predatory rattlesnake venoms has been shown to not result from modifications of targeted blood proteins, but the evolution of serum proteins which bind the toxins and prevent their pathophysiological coagulotoxic actions [15].

Australia is well known for its extraordinary diversity of reptilian fauna, which includes over 200 species of snakes from six families and over 800 species of lizards across seven families [16]. In particular, the Australian continent also represents one of the only places in the world which possesses more venomous (approximately 150) than non-venomous (approximately 70) snake species [16]. Due to the extreme diversity of reptiles in Australia, it is unsurprising that reptiles represent the primary prey for the majority of Australian elapids. Species within the genera *Austrelaps*, *Cacophis*, *Cryptophis*, *Demansia*, *Drysdalia*, *Furina*, *Hemiaspis*, *Pseudechis*, *Suta* and *Vermicella* are all known to either primarily or exclusively feed upon reptiles [17,18,19,20].

Australian elapid snakes are unique relative to African, American, and Asian elapids in being potently procoagulant. This is due to the blood-clotting enzyme Factor Xa being recruited for use as a toxin at the base of the Australian snake radiation [21]. FXa has a rate-limiting step in that it must bind in a 1:1 ratio with Factor Va in order to form a functional prothrombinase complex. This catalytic limitation was removed in the last common ancestor of the *Oxyuranus/Pseudonaja* clade when FVa was recruited as another venom component [22]. The differential action of these procoagulant venoms against the plasmas of taxonomically distinct lineages remains poorly investigated, with the effects primarily determined by testing only upon mammalian plasma or mammalian clotting factors [21,22,23,24,25]. One study which compared the action of tiger snake venom between amphibian and avian prey did not find significant differences, but also used non-native species as models for comparison (cane toad and domestic chicken) [23]. A study which compared human effects to animal models found wildly variable results, calling into question the suitability of comparing the activity as well as the potency of snake venoms upon animal plasma to human plasma [26].

Only one study has been undertaken investigating the in vivo lethality of a range of Australian snake venoms upon prey classes which yielded intriguing results, such as species of *Ctenotus* and *Egernia* skinks surviving high venom doses of *Notechis*, *Pseudonaja* and *Acanthophis* venom [27]. Another study examining the ability of Australian elapid snakes to be resistant to the procoagulant effects of their own venom found that *Notechis scutatus* and *Tropidechis carinatus* FXa venoms did not induce coagulation in *Notechis scutatus* or *Pseudonaja textilis* plasma, although were effective at clotting the plasmas of the outgroup species (western diamondback rattlesnake (*Crotalus atrox*) and reticulated python (*Malayopython reticulatus*)) [28]. The plasma of the FXa:FVa species *P. textilis* was not clotted by *N. scutatus* or *T. carinatus* venoms, whereas *P. textilis* was able to clot its own plasma, thus demonstrating that auto-resistance is not always present [28].

Monitor lizards are generalist predators which have been routinely observed feeding upon snakes, including highly venomous species such as black snakes (*Pseudechis* ssp) and brown snakes (*Pseudonaja* ssp) (Fry, personal observations). Monitor lizards are anecdotally said to be resistant to snake venom effects, although no studies have investigated the effect of coagulotoxins upon monitor lizards; recent research has also shown that the Australian *Varanus* spp. lack any modifications in their nicotinic acetylcholine receptor orthosteric site, which would confer resistance to α-neurotoxins [29]. This prior work has suggested that the lack of resistance towards neurotoxins in *V. giganteus* and *V. varius* is due to the protection afforded by the thick scales which contain protective pieces of bone (osteoderms), as well as efficient prey handling which allows them to successfully predate upon elapids.

This study aimed to fill the knowledge gap concerning the actions of procoagulant Australian elapid snake venoms against sympatric prey and also against sympatric predators. We investigated the in vitro coagulotoxic activity of specific Australian elapid species by determining the effects of their venom upon the clotting ability of plasma from sympatric species which may represent either prey (skinks in the *Tiliqua* genus) or predators (monitor lizards in the *Varanus* genus). The results also shed light in differences in fundamental clotting parameters between the different lizard species included in this study.

## 2. Results

The plasma of *T. scincoides* displayed only a slow response in clotting time in the presence of kaolin, indicating a relatively weak intrinsic pathway, whereas *T. rugosa* showed no clotting in the presence of kaolin (Table 1). *T. scincoides* also had no observable spontaneous clotting time, which is comparable to amphibian plasma (Table 1). The intrinsic pathway was shown to be active in all three species of monitor lizards, with *V. mertensi* plasma having the fastest clotting plasma in the presence of kaolin. *Varanus giganteus* plasma showed a slow spontaneous clotting time, whereas the plasma of *V. varius* had a spontaneous clotting time similar to that of human plasma. Clot strength was variable across all taxa included in this study, with the plasma of *V. varius* showing the strongest clot strength after activation of the intrinsic pathway relative to all other species (Table 1).

**Figure 1:**
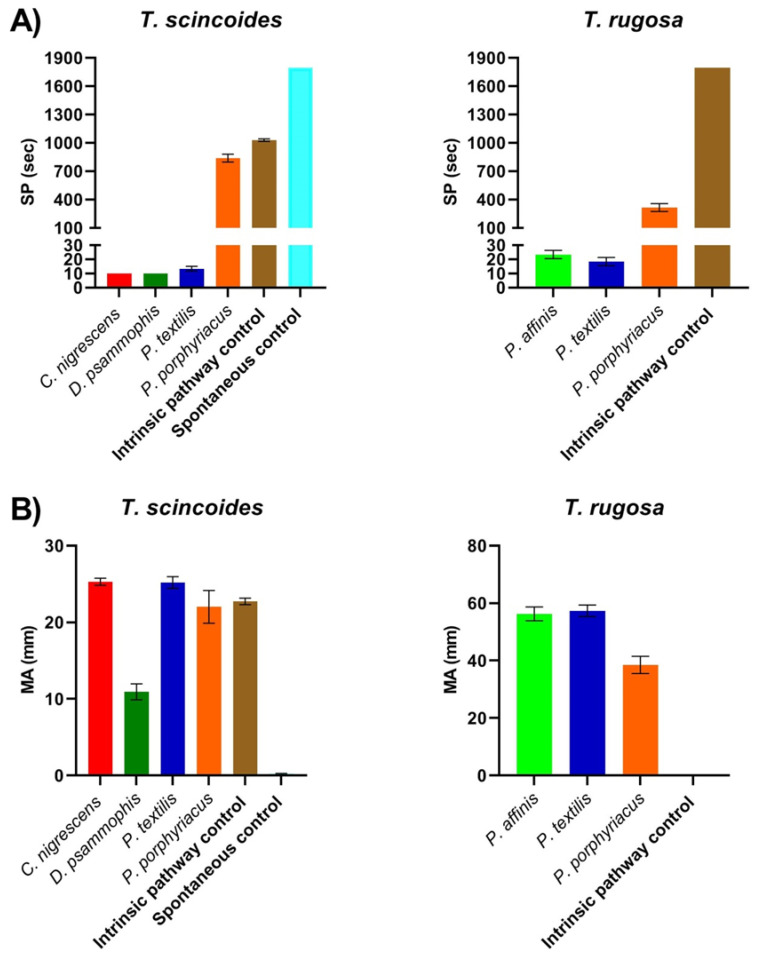
Coagulotoxic activity of Australian elapid venoms upon the plasma of the sympatric prey skink lizard species *T. rugosa* and *T. scincoides*. (**A**) Time taken until clot formation represented by SP (split point), which equals time taken until the clot begins to form (s). (**B**) Strength of clot formation represented by MA (maximum amplitude) which equals maximum clot strength (mm). All data points are *N* = 3 mean and standard error of the mean.

Venom from the species *C. nigresecens*, *D. psammophis* and *P. textilis* all showed potent procoagulant activity upon the clotting time of *T. scincoides* plasma (Figure 1A). *Pseudechis porphyriacus* venom did not show significant procoagulant activity upon the clotting time of *T. scincoides* plasma (Figure 1A). *Pseudonaja affinis* and *P. textilis* venom also showed potent procoagulant activity upon the plasma of *T. rugosa*, whereas the procoagulant activity of *P. porphyriacus* was considerably weaker upon *T. rugosa* plasma, although to a lesser extent than seen for *T. scincoides* plasma (Figure 1A). All venoms induced clots of considerable strength upon *Tiliqua* plasma, with the exception of *D. psammophis* venom upon *T. scincoides* plasma, which was approximately half the strength of the clot induced by the intrinsic pathway control as well as the other venoms (Figure 1B).

**Figure 2:**
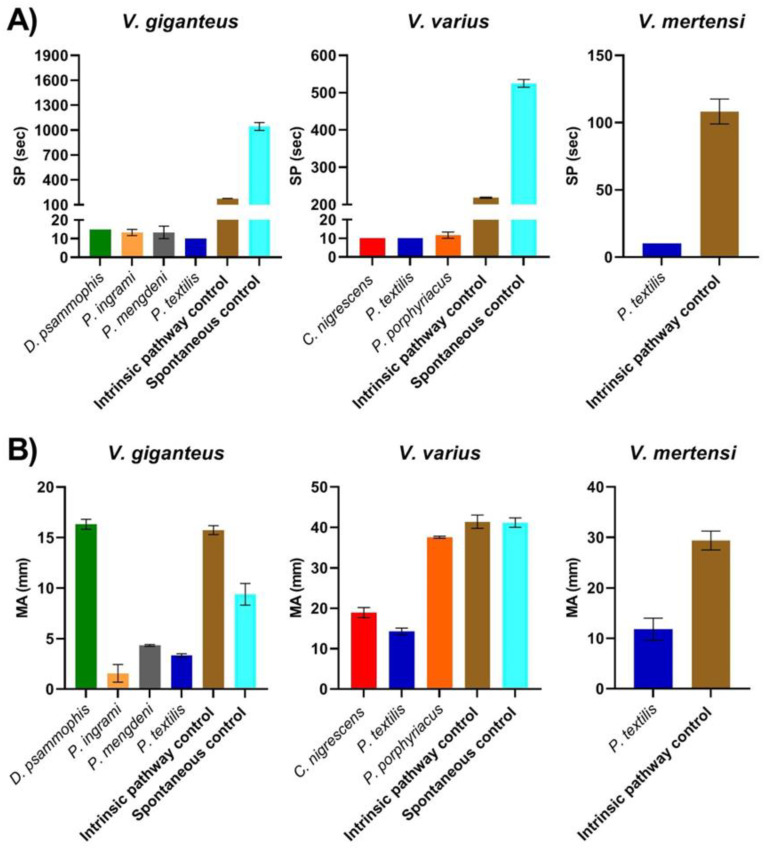
Coagulotoxic activity of Australian elapid venoms upon sympatric predatory varanid lizard species *V. giganteus*, *V. varius* and *V. mertensi* plasma. (**A**) Time taken until clot formation represented by SP (split point), which equals time taken until the clot begins to form (s). (**B**) Strength of clot formation represented by MA (maximum amplitude), which equals maximum clot strength (mm). Data points are *N* = 3 mean and standard error of the mean, except for the effect of *C. nigrescens* venom upon *V. varius* plasma and *P. textilis* venom upon *V. mertensi* plasma, which were *N* = 2 mean and standard error.

The venom of *D. psammophis*, *P. ingrami*, *P. mengdeni* and *P. textilis* all showed potent procoagulant activity upon the clotting time of *V. giganteus* plasma (Figure 2A). *Cryptophis nigrescens*, *P. textilis* and *P. porphyriacus* venom all had potent procoagulant activity upon *V. varius* plasma, as did *P. textilis* venom upon *V. mertensi* plasma (Figure 2A). The ability for venoms to induce clots of strength equal to that of the intrinsic pathway control was highly variable on *Varanus* plasma. All *Pseudonaja* venoms, as well as *C. nigrescens* venoms, induced clots much weaker than the controls on *Varanus* plasma (Figure 2B). However, *D. psammophis* and *P. porphyriacus* venom induced clots which were comparable in strength to the controls (Figure 2B).

## 3. Discussion

There were substantial differences in the fundamental clotting parameters between the lizard species examined in this study. Kaolin is routinely used to trigger the intrinsic pathway, but the lizard species were highly variable in the response of their intrinsic pathway (Table 1). The response by *T. scincoides* was weak, and *T. rugosa* was not triggered at all. In contrast, kaolin induced the intrinsic pathway of all *Varanus* species at a rate comparable to that of human plasma. Similarly spontaneous clotting was also highly variable between species (Table 1). *Tiliqua scincoides* plasma did not spontaneously clot, *V. giganteus* exhibited a slow spontaneous clotting time, and *V. varius* had a spontaneous clotting time comparable to that of humans.

*C. nigrescens, D. psammophis* and all *Pseudonaja* venoms rapidly induced clot formation in *Tiliqua* plasma (Figure 1A). Although as adults the diet of *Pseudonaja* is primarily mammalian, they do predate upon lizards, and both *T. rugosa* and *T. scincoides* have been recorded in the diet of *P. textilis* [30,31]. *Demansia psammophis* venom, although still exerting a rapid procoagulant effect upon the plasma of *T. scincoides,* formed comparably weaker clots than the other venoms (Figure 1). This was not a limitation of the FXa venom phenotype, because the other FXa venom tested (*C. nigrescens*) formed clots at similar speeds but were over twice as strong. It is important to note that although *D. psammophis* is a skink specialist and sympatric to *T. scincoides*, even juvenile *T. scincoides* would likely be too large to predate upon, and potentially the coagulation factors present in *T. scincoides* plasma are significantly divergent to the smaller skink species *D. psammophis* favours, leading to the variation in clot strength.

The most notable result observed when assessing the plasma of *Tiliqua* species was the weak action of *P. porphyriacus* venom (Figure 1). The procoagulant activity of *P. porphyriacus* was much weaker upon *Tiliqua* plasma (Figure 1A) than was observed for *V. varius* plasma (Figure 2A) and the previously determined effects upon human plasma [21]. One-way ANOVA with multiple comparisons confirmed a statistically significant difference between mean clotting time induced by the venom of *D. psammophis* (*p* < 0.0001), *C. nigrescens* (*p* < 0.0001) and *P. textilis* (*p* < 0.0001) in comparison to *P. porphyriacus* venom. Analysis also confirmed the statistically significant difference that the procoagulant activity of *P. porphyriacus* was significantly weaker upon *T. rugosa* plasma compared to *P. affinis* (*p* < 0.0001) and *P. textilis* (*p* < 0.0001). These results are highly suggestive of venom resistance in *Tiliqua* plasma towards the procoagulant toxins present in the venom of *P. porphyriacus*. In contrast, both *Tiliqua* species were much more strongly affected by small sympatric species (*C. nigrescens*, *D. psammophis*) that share the FXa venom phenotype, or venoms which were of the FXa:FVa venom phenotype (*Pseudonaja* venoms) (Figure 1). The venoms of both *C. nigrescens* and *D. psammophis* were still potently procoagulant and also possess the FXa venom phenotype; therefore, this indicates that a serum factor which specifically binds to *P. porphyriacus* toxins may be present in the plasma rather than modifications to *Tiliqua* coagulation factors causing the observed resistance.

*Pseudechis porphyriacus* are sympatric to *T. scincoides*, as well as other *Tiliqua spp*., across a large extent of their distribution [16,17]. Despite *T. scincoides* being a slow-moving, vulnerable prey-item, they are recorded as infrequent prey of *P. porphyriacus* [17]. This is further suggestive of the evolution of resistance in *T. scincoides* against *P. porphyriacus* venom. *Tiliqua rugosa* was also less affected by *P. porphyriacus* venom than other procoagulant venoms, perhaps indicating that a recent common ancestor developed resistance which has been retained to a greater extent by *T. scincoides*. Evidence which further supports that the slower action of *P. porphyriacus* is not a general limitation of this venom against lizards, but resistance exerted by *Tiliqua* species, is the rapid procoagulant effect *P. porphyriacus* venom exerted upon *V. varius* plasma (Figure 2A), in addition to studies which show that reptiles represent a large proportion of their dietary composition. This supports the hypothesis and evidence that *Tiliqua* have developed resistance towards the procoagulant toxins present within *P. porphyriacus* venom, which represent the toxins responsible for rapid prey subjugation in *P. porphyriacus* venom. This observed resistance may be partially responsible for the ecological success of *Tiliqua* and their extremely widespread distribution across the Australian continent. Future work should endeavour to investigate further the mechanisms behind the apparent venom resistance of *Tiliqua* plasma, in particular *T. scincoides*, towards *P. porphyriacus* venom.

The procoagulant activity of all Australian elapid species was shown to be fast-acting upon the plasma of all three *Varanus* species included in this study (Figure 2A), but with extreme variability in the strength of the clot formed (Figure 2B). In comparison to the intrinsic pathway control, the FXa venom phenotype representatives *D. psammophis* (*p* = 0.9833) and *P. porphyriacus* (*p* = 0.1735) produced strong clots in the plasma of *V. giganteus* and *V. varius*, respectively, whereas the clot induced by *C. nigrescens* (*p* < 0.0001) was significantly weaker on *V. varius* plasma (Figure 2B). In contrast, with respect to the intrinsic pathway control, the FXa:FVa phenotype venoms *P. ingrami* (*p* < 0.0001) and *P. mengdeni* (*p* < 0.0001) produced very weak clots in *V. giganteus* plasma, as did *P. textilis* (*p* < 0.0001) for *V. varius* (Figure 2B). The weak clots formed by the FXa:FVa venoms were not a lizard specific action though, because these venoms formed strong clots in the skink plasmas (Figure 1B). However, whereas *V. giganteus* and *V. varius* take venomous snakes as a part of their diet, *V. mertensi* does not [32,33], and the FXa:FVa venom phenotype also did not form strong clots in *V. mertensi* plasma despite being rapidly procoagulant. The generalised formation of weak clots by the FXa:FVa venoms on *Varanus* leads to the following competing hypotheses. The most likely is that the long period of divergence of nearly 200 million years ago between skinks and varanid lizards [34] has, through evolutionary drift, resulted in prothrombin sequences that are distinct enough from each other that the FXa:FVa venom phenotype does not cleave at the same site, resulting in divergent forms of thrombin produced, which, in turn, differentially cleave fibrinogen, to form divergent fibrin clots. Future work to test this hypothesis would require ascertaining the full-length sequences of prothrombin zymogens from *Tiliqua* and *Varanus*, ascertaining the cleavage site upon prothrombin and, in turn, ascertaining the cleavage site of the thrombin formed upon the corresponding fibrinogen chains.

Alternatively, resistance to *Pseudonaja* venoms is a trait common to all Australian varanid species because this genus is widespread and is fed upon by many varanid species in Australia. Thus, the recent specialisation of *V. mertensi* for an aquatic lifestyle has not led to a loss of this trait, which is consistent with it being a close relative of *V. giganteus* [35]. However, the venom of *C. nigrescens*, which possesses the FXa phenotype, also induced a weak clot which indicates that the formation of weak clots is not restricted to FXa:FVa venoms. Additionally, although weaker clots were formed, the speed of action was still extremely fast and weaker clots may still induce lethal stroke. Thus, in the case of varanid lizards, the weaker clots formed are unlikely to be suggestive of resistance, but this must be regarded tentatively as a possibility until future work is conducted. If the hypothesis that the difference in clot formation is due to simple drift in the prothrombin zymogens, this suggests that the regular predation upon venomous snakes by Australian varanid species is accomplished by the mechanical protection afforded by the thick scales which are reinforced by osteoderms, combined with the high intelligence of these lizards and efficient prey handling. Thus, the belief that Australian monitor lizards are resistant to venomous snakes must be regarded as unresolved at this point in time, but the current evidence supports that they are not resistant to sympatric snake venoms.

## 4. Conclusions

In conclusion, we identified significant variability in the clotting time and clot strength of plasma from five species of Australian lizard, as well as identified notable variations in the coagulotoxic activity of sympatric venomous elapid species. Significant variation in the ability of both venom phenotypes (FXa and FXa:FVa) to induce a strong clot was observed across both *Tiliqua* and *Varanus* venoms. Furthermore, our results strongly suggest that *Tiliqua* species are resistant to the procoagulant toxins of the sympatric *P. porphyriacus*, because the venom action was greatly delayed relative to other venoms, including those sharing the FXa venom phenotype. In contrast, the varanid lizards did not delay the venom action of the *Pseudonaja* species upon which they feed, but with the strength of clot being significantly diminished. This may be weakly suggestive of some level of resistance, but the morphological and behavioural characteristics more strongly support Australian varanid lizards not possessing resistance to the activity of the venom from the snakes which they predate upon. At this time, the only varanid lizard with documented resistance to snake venom remains that of *V. exanthematicus* [29]. Thus, this study not only sheds new light upon predator–prey interactions, but also identifies a new case of venom resistance which warrants further investigation to determine the mechanisms behind the resistance of *Tiliqua* plasma towards *P. porphyriacus* venom.

## 5. Materials and Methods

### 5.1. Venoms and Reagents

All venom work was conducted under University of Queensland Biosafety Committee Approval # IBC/134B/SBS/2015 and venoms were individually sourced from the long-term cryogenic research collection of the Venom Evolution Lab, except for *P. ingrami* and *P. textilis*, which were pooled samples sourced from Venom Supplies, South Australia. Tested were the FXa venoms *Cryptophis nigrescens* (Bunya, Queensland, Australia), *Demansia psammophis* (Cedar Creek, Queensland, Australia), and *Pseudechis porphyriacus* (Unknown locale). Additionally, tested were the FXa:FVa venoms *Pseudonaja affinis* (Esperance, Western Australia), *Pseudonaja aspidorhyncha* (Dubbo, New South Wales, Australia), *Pseudonaja ingrami* (unknown locale), *Pseudonaja mengdeni* (Alice Springs, Northern Territory, Australia), and *Pseudonaja textilis* (Barossa Valley, New South Wales, Australia). Original venom extractions were flash-frozen, lyophilized, and stored at −80 °C. Using a NanoDrop2000 UV-Vis Spectrophotometer (Thermofisher, Sydney, NSW, Australia) to determine venom concentrations, venom working stock solutions were made at a concentration of 1 mg/mL in 50% glycerol and 50% double-deionised water (DDH_2_O) and stored at −20 °C until required to preserve enzymatic activity. Calcium chloride (Stago catalog #00367), kaolin (Stago catalog #00597), phospholipid (Stago catalog #00597) and Owren-Koller buffer (Stago catalog #00360) were supplied by Stago (Stago, Paris, France).

### 5.2. Plasma Collection

All plasma work was conducted under University of Queensland Biosafety Committee Approval # IBC/149B/SBS/2016 and Animal Ethics Committee Approval #2020/AE000324. All reptile plasma was collected from captive, healthy adult individuals during routine veterinary health checks. Plasma was collected from the caudal vein and pooled from multiple individuals when possible. *Tiliqua rugosa* plasma was collected from seven individuals, *T. scincoides* plasma was collected from nineteen individuals, *V. mertensi* plasma was available only from a single individual, *V. giganteus* plasma was from two individuals, and *V. varius* plasma from three individuals. *Gallus gallus* plasma was pooled from nine individuals and collected at the University of Queensland Gatton Campus. *Rhinella marina* plasma was a pooled sample from thirty-five wild-caught individuals. *Rattus norvegicus* plasma was supplied by Animal Resources Centre Western Australia, and was a pooled sample from twenty-five individuals. Pooled human plasma was supplied by the Australian Red Cross Blood Service and stored at −80 °C.

### 5.3. Thromboelastography Assays

Thromboelastography was utilised to test the in vitro viscoelastic clotting properties of plasma in the absence and presence of venom. Thromboelastography assays were adapted and utilised from previously published protocols [36,37,38]. For all assays, 7 µL of either venom (1 mg/mL) or control reagent was added to 72 µL calcium, 72 µL phospholipid, 20 µL Owren Koller buffer and 189 µL plasma. To determine the natural activation of the clotting cascade via the intrinsic pathway for each species’ plasma, positive controls were conducted using 7 µL of kaolin in replacement of venom for this assay. To determine the natural spontaneous clotting time of each species plasma, controls were conducted using 7 µL of 50% glycerol and 50% DDH_2_O in the replacement of venom. Where possible, only sympatric species of venom were tested upon plasma, although *D. psammophis* venom was used upon the plasma of *V. giganteus* because venom was not available from any species of *Demansia* sympatric to *V. giganteus*.

### 5.4. Data Analysis

All investigations were conducted in triplicate. All data in this study were analysed using Prism 9.0 software (GraphPad Software Inc., La Jolla, CA, USA). One-way ANOVAs with multiple comparisons were conducted to assess statistical significance throughout the study. Potent procoagulant activity was considered in this study as venom activity which caused clotting to occur in less than 50 s. All raw data is available in Supplementary File S1. 

## Figures and Tables

**Table 1:** Comparative clotting times and clotting strengths for vertebrate plasma. SP (split point) equals time taken until the clot begins to form (s). MA (maximum amplitude) equals maximum clot strength (mm).

	Coagulation via Intrinsic Pathway Activation		Coagulation via Spontaneous Clotting	
Species	SP (s)	MA (mm)	SP (s)	MA (mm)
*Tiliqua scincoides*	1028.33 ± 13.02	22.73 ± 0.42	*	*
*Tiliqua rugosa*	*	*	NA	NA
*Varanus giganteus*	171.67 ± 6.67	15.73 ± 0.44	1045 ± 48.05	9.4 ± 1.07
*Varanus mertensi*	108.33 ± 9.28	29.4 ± 1.86	NA	NA
*Varanus varius*	218.33 ± 1.67	41.4 ± 1.65	525 ± 10.41	41.2 ± 1.16
*Rhinella marina*	65 ± 2.89	7.4 ± 0.12	*	*
*Gallus gallus*	166.67 ± 3.33	44.2 ± 1.36	188.33 ± 4.41	39.03 ± 0.45
*Rattus norvegicus*	48.33 ± 3.33	19.03 ± 1.3	135 ± 5	22.57 ± 2.55
*Homo sapiens*	173.33 ± 1.67	17.67 ± 0.35	646.67 ± 24.55	20.2 ± 0.06

* Represents no clot was formed during the 30 min running time of the assay. NA represents tests which were not conducted due to insufficient plasma stocks.

## Data Availability

All data are available within the supplementary files.

## Supplementary Materials

The following are available online at https://www.mdpi.com/article/10.3390/toxins13090590/s1, Supplementary File S1 contains all raw data for this study.
